# Inter-scan Reproducibility of Cardiovascular Magnetic Resonance Imaging-Derived Myocardial Perfusion Reserve Index in Women with no Obstructive Coronary Artery Disease

**DOI:** 10.19080/CTCMI.2018.02.555587

**Published:** 2018-02-23

**Authors:** Ahmed Al-Badri, Janet Wei, Sofy Landes, Manish Motwani, Galen Cook-Wiens, Michael D Nelson, Puja K Mehta, Chrisandra Shufelt, Behzad Sharif, Debiao Li, Daniel S Berman, Louise EJ Thomson, C Noel Bairey Merz

**Affiliations:** 1 Cedars Sinai Heart Institute, USA; 2 Biostatistics and Bioinformatics Research Center, Cedars-Sinai Medical Center, USA; 3 S Mark Taper Foundation Imaging Center, Cedars-Sinai Medical Center, USA

**Keywords:** Cardiac magnetic resonance imaging, Myocardial perfusion reserve index, Inter-scan, Reproducibility

## Abstract

**Background:**

Cardiovascular magnetic resonance imaging (CMRI) derived myocardial perfusion reserve index (MPRI) has recently been shown to detect coronary microvascular dysfunction (CMD) in women with signs and symptoms of ischemia and no obstructive coronary artery disease (CAD). The aim of this study was to determine the inter-scan reproducibility of MPRI in this patient group in order to assess its diagnostic robustness in serial scans and assess its utility as a marker of potential therapies for CMD.

**Methods:**

Rest/stress perfusion CMR was performed at 1.5T using a standardized protocol in 17 women with signs and symptoms of ischemia and no obstructive CAD on two separate days (within 90 days of each other). The same pharmacological stress agent (adenosine/regadenoson) was used for both scans. MPRI was calculated from time-intensity curves of the whole myocardium and blood pool at stress and rest. One experienced observer, blinded to clinical data, performed all measurements. Intra-class correlation coefficients (ICC), coefficient of variation (CoV), and Bland-Altman plots were determined.

**Results:**

Mean age was 53±10 years old and BMI 28±7 kg/m2; 47% had hypertension, 4% diabetes, 9% hyperlipidemia and 10% family history of CAD. Mean MPRI for the 17 women was higher for scan 2 compared to scan 1 (1.98±0.3 vs. 1.65±0.78, respectively, p<0.001); and this relationship persisted even when corrected for resting rate pressure product (RPP) (2.42±0.81 vs. 1.97±0.92, respectively, 0.002), The mean bias for MPRI between sequential scans was 0.34 (95% CI: 0.18 to 0.49, limits of agreement: −0.31, 0.98 and when corrected for resting RPP it was 0.45 (95% CI: 0.21 to 0.68, limits of agreement: −0.52, 1.41), ICC and CoV also indicated modest inter-scan reproducibility (ICC 0.57; CoV 20.3%), but both measures were comparable to values seen in prior studies in CAD populations and healthy volunteers.

**Conclusion:**

Inter-scan reproducibility of CMRI-derived MPRI in women with suspected CMD is modest, with relatively wide limits of agreement. This variability is similar to that seen in other populations, suggesting that some caution must be exercised when using absolute MPRI cut-offs in isolation for the diagnosis of CMD or repeated measures of MPRI to track response to therapy. Additional work is ongoing to improve reproducibility from both biological and technological standpoints.

## Introduction

First-pass stress perfusion cardiac magnetic resonance imaging (CMRI) can detect vasodilator stress-induced myocardial hypo-perfusion in patients with coronary artery disease (CAD) with high sensitivity and specificity [1,2]. Women with signs and symptoms of ischemia but no obstructive CAD are an increasingly recognized patient group requiring more investigational study to understand the best diagnostic and management strategies. These patients often have coronary micro vascular dysfunction (CMD) which is associated with cardiovascular adverse outcomes [1,2]. Invasive coronary reactivity testing is the reference standard for diagnosing CMD using different vasoactive medications to evaluate the endothelial and non-endothelial-dependent coronary function. Increased rates of cardiac death, stroke, and new onset heart failure have been observed during 4.5 years follow up among women with reduced invasive coronary flow reserve (CFR) to adenosine [3].

CMRI is noninvasive imaging technique that can be used to diagnose CMD through assessment of rest and stress myocardial perfusion [4]. Semi-quantitative analysis of the first-pass perfusion CMRI data can be used to calculate myocardial perfusion reserve index (MPRI), which is an indexed ratio of perfusion time intensity curves, as a measure of response to vasodilator stress, while positron emission tomography (PET) has a well-established evidence-base for robust non-invasive CFR assessment. CMRI has the advantage of the lack of radiation which is particularly relevant in the predominantly young women who comprise this patient group. This advantage would be of particular importance for the use of serial imaging to monitor treatment response. In the context of serial studies, the inter-scan reproducibility of CMRI derived MPRI measurement needs to be established, so that differences between groups or values obtained in individuals can be defined as being real or potentially due to known variation in measurement. Therefore, the aim of this study was to assess the inter-study agreement of semi-quantitative MPRI in women with signs and symptoms of ischemia and no obstructive CAD undergoing serial stress perfusion CMRI.

## Methods

### CMRI procedures

Seventeen women underwent 2 serial rest/stress perfusion CMRI scans within 90 days of each other as part of their participation in the RWISE study [5]. The study was an IRB approved clinical trial under the care of the Women’s Heart Center, Cedars-Sinai Heart Institute, Los Angeles, California and university of Florida, Gainesville, FL (NCT01342029), Accordingly, all subjects had been referred for evaluation of signs and symptoms of ischemia with no obstructive CAD (defined as <50% luminal diameter stenosis of epicardial coronary artery on invasive coronary angiography). Subjects with significant CAD (epicardial artery stenosis ≥50%), coronary artery anomalies, and visible coronary vasospasm during angiography or bridging were excluded.

Therefore, data from a total of 34 scans performed were available for analysis. The paired scans were performed with the same vasodilator stress agent i.e. using adenosine or regadenoson. All studies were performed between May 2011 and September 2015. All subjects had evidence of CMD confirmed by either invasive coronary reactivity testing (defined as CFR <2.5, or no dilatation [≤0% change] in response to acetylcholine) [6] as part of clinical care or stress CMRI (defined as MPRI<2.0), All vasoactive medications were stopped at least 24 hours before CMR testing as per protocol [5]. The rest/stress CMRI was performed following the previously published protocol [4].

### CMR quantitative analysis

**Figure 1:**
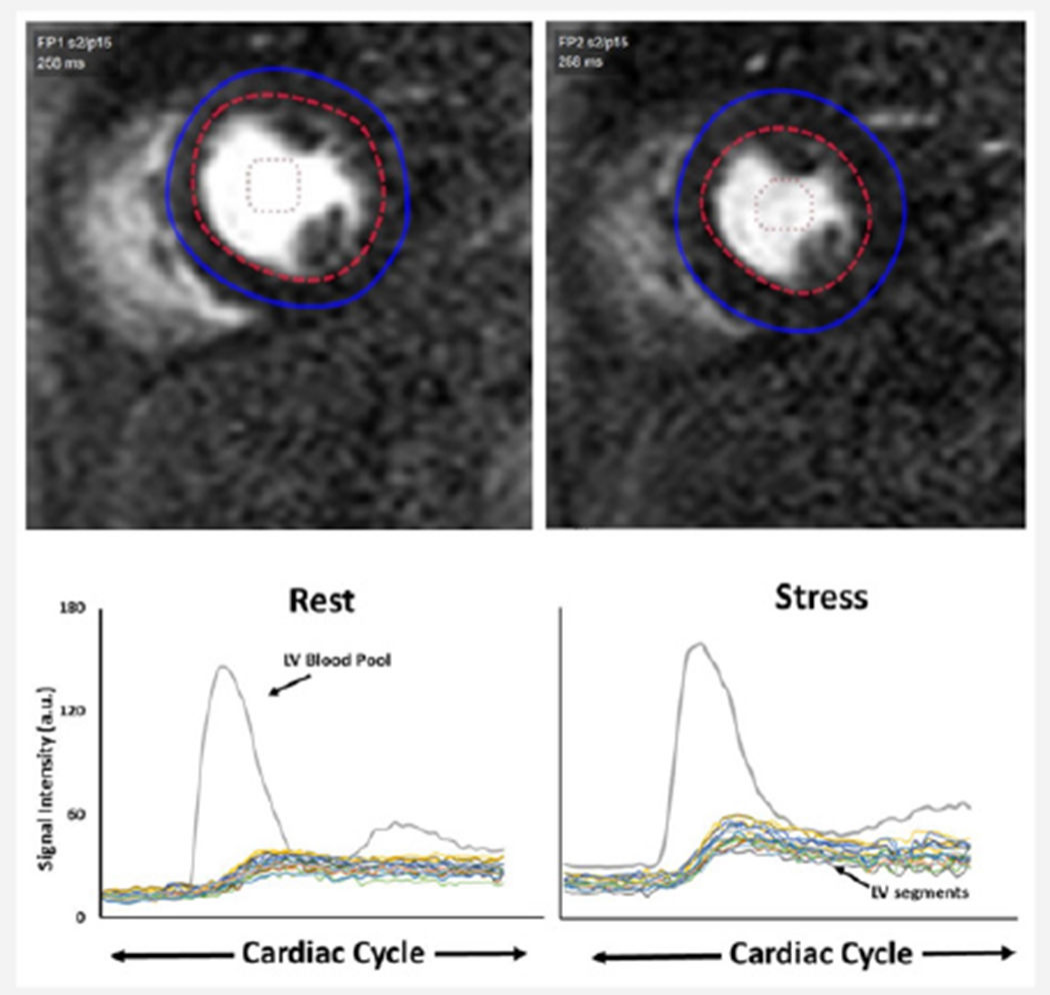
Cardiac Magnetic Resonance Imaging (CMRI) at rest and first pass myocardial perfusion images with corresponding intensity-over-time curves. Signal intensity for the mid-left ventricular cavity (green curve) and for the 6-segments myocardial first pass increment in signal.

CMRI data were interpreted by one expert reader experienced in performance and interpretation of CMRI (L.E.J. Thomson) blinded to clinical data. Semi- quantitative analysis of the first pass perfusion images was performed using CAAS MRV 3.3 software (Pie Medical Imaging B.V., Netherlands). The endocardial and epicardial contours were manually defined and adjusted, frame by frame if needed, to optimize sampling of the myocardium. Care was taken to exclude blood pool activity and to exclude any linear dark rim artifact at the LV cavity/endocardial border. The LV cavity region of interest was manually adjusted to include the region of maximal signal intensity within the cavity and to exclude papillary muscle. The reader manually defined the starting point (T0 cycle) and the ending point (T end) of time intensity curves. TO was set at the baseline point immediately prior to the upslope and T end was placed at the point where myocardial peak intensities were reached (Figure 1), The ratio of the maximum upslope of the selected curve, which corresponds to the specific myocardial segment, over the maximum upslope of the LV cavity curve, gives the relative upslope (RU). MPRI is then calculated by RU at stress divided by RU at rest. Data is generated by the software for subendocardial, subepicardial and transmural MPRI using standard AHA myocardial segmentation (16 segments due to absence of data for an apical segment) [7].

### Statistical analysis

Variables were summarized by means and standard deviations, or counts and percentages if categorical. The ICC was calculated as the proportion of between subject variance from a linear regression model with only a fixed intercept effect and random subject intercepts. Coefficient of variation is reported as a percent of the standard deviation divided by the mean. An analysis of measurement agreement was carried outas described in Bland and Altman [8]. Significance for hypothesis tests was set at a level of 0.05. All analyses were done using SAS version 9.3 (SAS Institute, Cary, NC) and R software. The MPRI was corrected to rate pressure product: Corrected MPRI: MPRI/rest RPP *104 if there is variability in heart rate and systolic blood pressure between scans for each subject [9].

## Results

Subject characteristics are shown in Table 1 for the whole group. Mean age was 53±10 years old and BMI 28±7kg/m2; 47% had hypertension, 4% diabetes, 9% hyperlipidemia and 10% family history of CAD. No significant complications occurred and all subjects completed the imaging protocol. The MPRI results are shown in Table 2. MPRI data is presented for the transmural (16 segment mean) as well as individual slices for both subendocardial and subepicardial regions. Inter-scan reproducibility of the left ventricular MPRI calculated by ICC and expressed as CoV.

**Figure 2:**
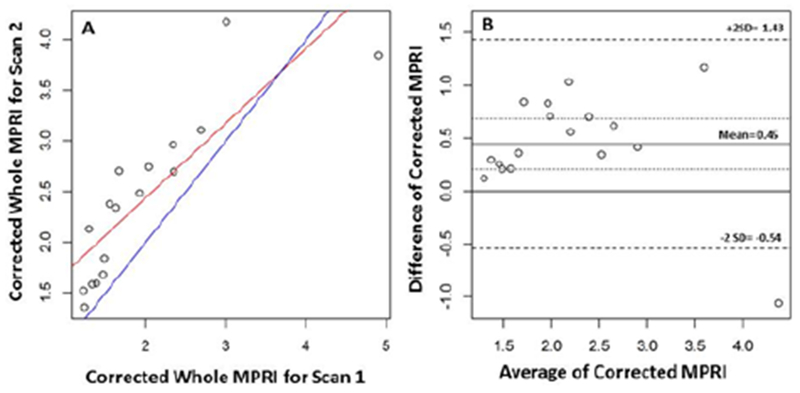
Agreement of Whole Myocardial Perfusion Reserve Index (MPRI) between Scan 1 and Scan 2. Linear regression graph (A) and Bland-Altman plot (B).

**Figure 3:**
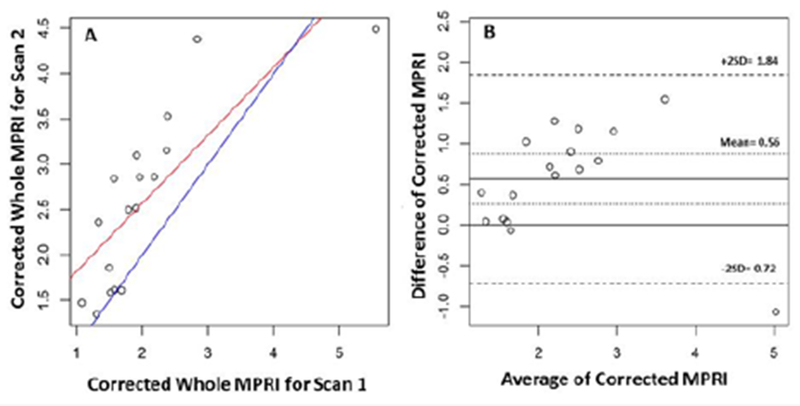
Agreement of Mid-ventricular Myocardial Perfusion Reserve Index (MPRI) between Scan 1 and Scan 2. Linear regression graph (A) and Bland-Altman plot (B).

Mean MPRI was higher for scan 2 compared to scan 1 (1.98±0.3 vs. 1.65±0.78, respectively, p<0.001); and this relationship persisted even when corrected for resting RPP (2.42±0.81 vs. 1.97±0.92, respectively, p<0.002). The average difference for MPRI between scans was 0.34 (95% CI: 0.18 to 0.49) and for resting RPP corrected MPRI it was 0.45 (95% CI: 0.21 to 0.68). The result of Bland-Altman analysis for transmural and mid-ventricular MPRI is shown in Figure 2 & 3, respectively. The limits of agreement for the 0.34 bias in MPRI reproducibility were (−0.31, 0.98) and for the 0.45 bias in resting RPP corrected MPRI reproducibility they were (−0.52, 1.41). The subendocardial MPRI for all slices was more reproducible (CoV 16.2) compared to mean whole MPRI (CoV 20.3) and mean subepicardial MPRI (CoV 20.7 %). There was a modest inter-scan ICC 0.57, 0.52 and 0.57 within transmural, subendocardial and subepicardial myocardial regions respectively. The CoV was 20% for the transmural MPRI.

## Discussion

In our study, first pass myocardial perfusion CMRI for semi-quantitative analysis of MPRI measurements on serial CMRI studies, using commercially available software, was shown to have modest inter-scan reproducibility. The inter-scan reproducibility of CMRI-derived MPRI in the novel population of women with suspected CMD was modest, with relatively wide limits of agreement. There is limited published data available for the reproducibility of serial myocardial perfusion measurements using CMRI. Inter-study reproducibility of CMRI has been reported for LV volumes, ejection fraction and mass [9–11], and there is limited data describing the reproducibility of semi-quantitative analysis of stress perfusion CMR. We have previously defined reproducibility of repeated MPRI calculation for CAAS MRV 3.3 software (Pie Medical Imaging B.V., Netherlands) and found intra-observer coefficient of variation 3.6%, inter-observer coefficient of variation 7.5% (12). The inter-scan reproducibility of CMRI myocardial stress perfusion has been reported using visual, semi-quantitative and fully quantitative approaches. Larghat et al. [12] studied the reproducibility of myocardial perfusion CMRI in 11 normal subjects (including 5 women) during adenosine stress and rest on 2 separate days with mean interscan delay of 84 days (range 7-280 days). Imaging was at 1.5T (Philips Medical Systems) and post processing used Q Mass 6.1.6, Medis. The coefficient of variation (CoV) was 19.4% for semi-quantitative inter-study comparison and 27% for fully quantitative analysis of myocardial perfusion reserve. Chih et al. [9] evaluated twenty subjects (10 with CAD and 10 controls at low Framingham risk of CAD, including 4 women). Imaging was at 1.5T (Philips Medical Systems) and post processing used Philips View Forum workstation. The CoV for visual (qualitative) segmental analysis of patients was 30.6%. The CoV for global MPRI was 23% for patients with CAD and 18% in the control group. They also reported software based reproducibility data with intra-observer CoV of 5.3%, inter-observer CoV 9.0%.

PET is a well-established method for non-invasive quantitation of myocardial flow and prior studies have shown variability in repeated measurement of hyperemic flow using absolute quantitative approaches with both radio labeled water and ammonia. Kauffman et al studied 21 normal volunteers (number of women not reported) with O labeled water with two assessments within one hour and reported repeatability coefficient (smallest real difference) for global measurement of coronary vasodilator reserve of 1.32 (33% of mean) [13]. Nagamachi et al. [14] used [13] N-ammonia PET with repeated measurements in volunteers (including 4 women) and reported variation in both rest and stress absolute flow measurements repeated same day (n=8) or different day (n=13). Differences were normalized to rate pressure product, with reported two day mean percentage difference in absolute vasodilator stress perfusion 10.3±10.5%. More recently, repeated measurement of coronary flow reserve using 82Rubidium PET reported a mean difference of −4.14±18% between first and second measures performed 60 minutes apart in 15 healthy volunteers (including 7 women) [15].

In our data, there were wide limits of agreement for serial MPRI measurement derived from CMR (Figure 2). This emphasizes the need for caution in over interpreting small changes in MPRI between repeated studies. There are multiple factors that potentially contribute to variation in myocardial perfusion reserve measurement. By protocol, the two scans were performed at the same time of day, with identical pre-test preparation (including medication and caffeine withdrawal). scanner hardware and software settings and pharmacologic stress agent. The influence of resting hemodynamic state was adjusted for in statistical analysis. Variation related to post processing of data was minimized by use of a single observer.

The main limitation of this study is the retrospective nature. Thus, there might be a change in subjects’ disease process, medications and clinical status between the scans. However, we tried to select subjects whose their medications and clinical status unchanged between the two scans. Due to the latter, small number of subjects were included in this analysis. Yet, we believe the study is adequately powered to examine test-retest reproducibility, our sample is larger than the prior reports, and represents a first evaluation in women with microvascular dysfunction. Another limitation of our study is the length of time between scans (90 days). However in previous reproducibility study evaluated myocardial perfusion index using SPECT, the repeat scan was performed 9-22 months after the first one. The study showed that myocardial perfusion index was highly correlated and reproducible [16]. While we detected only a modest difference between test visits, there was a bias to increase in MRRI on the second compared to the first visit. Patients were being clinically managed between the two visits, making it possible that the improvement bias reflects ‘real’ change, perhaps due to use of medications such as HMG-CoA reductase inhibitors, ACE inhibitors and angiotensin receptor blockers that could have influenced microvascular function but were not withdrawn prior to stress testing [17]. Our study did not include normal subjects, though this is more closely aligns with clinical practice as patients with normal test are less likely to undergo serial testing. We believe more work in this area is needed.

## Conclusion

Inter-scan reproducibility of CMRI-derived MPRI in women with suspected CMD is modest, with relatively wide limits of agreement. This variability is similar to that seen in other populations, and is comparable to PET, suggesting that some caution must be exercised when using absolute MPRI cut-offs in isolation for the diagnosis of CMD, or repeated measures of MPRI to track response to therapy. Additional work is ongoing to improve reproducibility and understand sources of variation of MPRI measurement from both biological and technological standpoints.

## Figures and Tables

**Table 1:** Demographic and Clinical characteristics (n=17).

Age (years)	53±10
BMI (kg/m^2^)	28±7
Hypertension	8 (47%)
Diabetes	4 (24%)
Hyperlipidemia	9 (53%)
Family history of premature coronary artery disease	10 (59%)
Current smoker	0%
Former Smoker	4 (24%)
**Symptoms**	
Typical angina	9 (53%)
Shortness of breath	13 (77%)
Palpitations	8 (47%)
Nausea	4 (23.5%)
**Medications**	
- Beta blockers	9 (53%)
- Calcium channel blockers	8 (47%)
- ACEI or ARB	(29%)
- Nitrates	13 (77%)
- HMG-CoA reductase inhibitors	10 (59%)
- Aspirin	14 (82%)
- Diuretic	2 (12%)

**BMI:** Basal metabolic index, ACEI: angiotensin-converting enzyme inhibitors, ARB: angiotensin receptor blockers

**Table 2:** Inter-scan reproducibility of post processing MPRI* (N=17).

	Scan 1	Scan 2	CoV	ICC
**All Slices**				
Mean whole MPRI segments 1-16	1.97±0.92	2.42±0.81	20.30%	0.57
Mean subendocardial MPRI segments 1-16	1.81±0.82	2.13±0.64	16.20%	0.52
Mean subepicardial MPRI segments 1-16	2.1±1.10	2.56±0.91	20.70%	0.56
**Basal Slices**				
Mean whole MPRI segments 1-6	1.98±0.90	2.31±0.82	15.70%	0.56
Mean subendocardial MPRI segments 1-6	1.88±0.82	2.06±0.70	9.70%	0.51
Mean subepicardial MPRI segments 1-6	2.02±1.01	2.51±0.96	21.80%	0.43
**Mid Slices**				
Mean whole MPRI segments 7-12	2.03±1.01	2.59±0.96	24.40%	0.55
Mean subendocardial MPRI segments 7-12	1.74±0.84	2.25±0.78	25.60%	0.47
Mean subepicardial MPRI segments 7-12	2.24±1.29	2.69±1.02	18.50%	0.60
**Apical Slices**				
Mean whole MPRI segments 13-16	1.94±1.02	2.39±0.75	17.40%	0.54
Mean subendocardial MPRI segments 13-16	1.88±0.96	2.13±0.62	7.80%	0.57
Mean subepicardial MPRI segments 13-16	1.99±1.13	2.54±0.96	21.70%	0.54

MPRI: Myocardial Perfusion Reserve Index, CoV: Coefficient of Variation, ICC: Intra-Class Correlation Coefficient

* MPRI was corrected to the resting rate pressure product

## Abbreviations:

CMRI: Cardiac Magnetic Resonance Imaging
CAD: Coronary Artery Disease
ICC: Intra-Class Correlation Coefficients
CoV: Coefficient of Variation
RPP: Rate Pressure Product
PET: Positron Emission Tomography
